# Planning and Providing Acute Stroke Care in England: The Effect of Planning Footprint Size

**DOI:** 10.3389/fneur.2019.00150

**Published:** 2019-02-27

**Authors:** Michael Allen, Kerry Pearn, Emma Villeneuve, Martin James, Ken Stein

**Affiliations:** ^1^NIHR CLAHRC South West Peninsula, University of Exeter College Of Medicine and Health, University of Exeter, Exeter, United Kingdom; ^2^Royal Devon and Exeter NHS Foundation Trust and the National Institute for Health Research (NIHR) Collaboration for Leadership in Applied Health Research and Care (CLAHRC) South West Peninsula, Exeter, United Kingdom

**Keywords:** stroke, health services research, health service planning, thrombolysis, genetic algorithm

## Abstract

**Background:** Guidelines in England recommend that hyperacute stroke units (HASUs) should have a minimum of 600 confirmed stroke admissions per year in order to sustain expert consultant-led services, and that travel time for patients should ideally be 30 min or less. Currently, 61% of stroke patients attend a unit with at least 600 admissions per year and 56% attend such a unit and have a travel time of no more than 30 min.

**Objective:** We have sought to understand how varying the planning and provision footprint in England affects access to care whilst achieving the recommended admission numbers for hyper-acute stroke care. We have compared two different planning footprints to national-level planning: planning using five NHS Regions in England, and planning using 44 Sustainability and Transformation Partnerships (STPs) in England.

**Methods:** Computer modeling and optimization using a multi-objective genetic algorithm.

**Results:** The number of stroke admissions between STPs varies by seven-fold, while the number of stroke admissions between NHS Regions varies by 2.5-fold. In order to meet stroke admission guidelines (600/year) for all units the maximum possible proportion of patients within 30 min would be 82, 78, and 72% with no boundaries to planning/provision, NHS Region boundaries, and STP boundaries (in these scenarios patients cannot move outside of their own STP or NHS Region). If STP or NHS Region boundaries are removed for provision of service (after planning is performed at these local levels), travel time is improved, but number of admissions to individual hospitals become significantly changed, especially at STP planning level where admission numbers per unit changed by an average of 204 (19%), and not all units maintained 600 admissions after removal of boundaries.

**Conclusion:** Planning and providing services at STP level could lead to sub-optimal service provision compared with using larger and more consistently populated planning areas.

## Introduction

Stroke is a major cause of burden on individuals and healthcare services. It was estimated that in 2010, there were 5.9 million deaths and 33 million stroke survivors (1). Eighty five thousand people are hospitalized with stroke each year in England, Wales and Northern Ireland (2). Over the last 25 years stroke was the leading cause of lost disability-adjusted life years, which combine mortality and disability burdens (3).

Centralization of stroke care in London, into large hyperacute stroke units (HASUs), where care is delivered by specialist stroke teams, has been shown to reduce mortality, reduce length of stay, increase thrombolysis rates and reduce long-term costs to the NHS (4, 5). As a consequence of these improved outcomes and reduced costs, the NHS in England has promoted the reconfiguration of stroke services across England with the aim that all stroke care is delivered in HASUs (6).

For acute stroke units, national guidelines recommend that each HASU should have at least 600 confirmed stroke admissions per year (7), with a recommendation that travel time should ideally be 30 min or less, and no more than 60 min (8).

The English NHS is facing increasing demand, bringing with it the need to continually improve and transform services. In 2015, NHS England, the statutory agency responsible for the provision of healthcare in England, announced 44 geographical areas that would build “Sustainability and Transformation Plans” (subsequently Partnerships; STPs, with some evolving into “Integrated Care Systems”) to deliver sustainable transformation in health and care outcomes between 2016 and 2021 (9, 10). STPs are a key footprint for planning of many acute services in England. In addition to STPs there are five NHS Regional teams which provide additional support and leadership in commissioning services. Each STP is assigned to one of the five regions.

We have previously described the use of a genetic algorithm to optimize the planning of the number and locations of acute stroke services (thrombolysis and thrombectomy) in order to maximize the proportion of patients with good access whilst meeting guidelines for the number of admissions (11, 12). In previous work we assumed no hard borders to planning or service provision. In this paper we investigate the effect of applying hard borders to planning provision, either around the five NHS Regions or the 44 STPs. We investigate how well a boarders system can perform compared to a border-less system, and we look at the effect of planning assuming borders but those borders then being ignored for provision of care.

We focus on HASUs providing thrombolysis as those are expected be the first point of access for acute stroke care.

## Methods

All data and optimization code used are available (see section Data Availability below). Detailed methodology is available in an on-line Appendix in Supplementary Material.

Detailed methodology has also previously been described (11).

### Data

Location data used Lower Super Output Areas (LSOA) across England. LSOAs are geographic areas with a population of ~1,500 and with an average distance of 2 km between nearest-neighbor LSOAs. There are 32,844 LSOAs in England, though we exclude the Isles of Scilly in this analysis. The home location of the person was taken as the population-weighted centroid of each LSOA (13). Travel times were based on the estimated fastest road travel times from the postcode closest to the population-weighted centroid of the parent LSOA to the postcode of the HASU. Travel times were estimated using Maptitude (www.maptittude.com) and MPMileCharter (http://www.milecharter.com) for normal road conditions without significant congestion.

Admissions for stroke per LSOA for 3 years 2014–2016 were obtained from NHS Hospital Episodes Statistics managed through Lightfoot Solutions (www.lightfootsolutions.com). We included 242,874 patients coded with an emergency admission of ischaemic or hemorrhagic stroke (primary diagnosis ICD-10 I61, I63, I64). Admission numbers per institution were obtained from the 2015/16 Sentinel Stroke National Audit Programme (SSNAP) for admissions (2). The location of 127 acutely admitting stroke units was taken from the 2016 SSNAP annual report (2).

**Figure 1:**
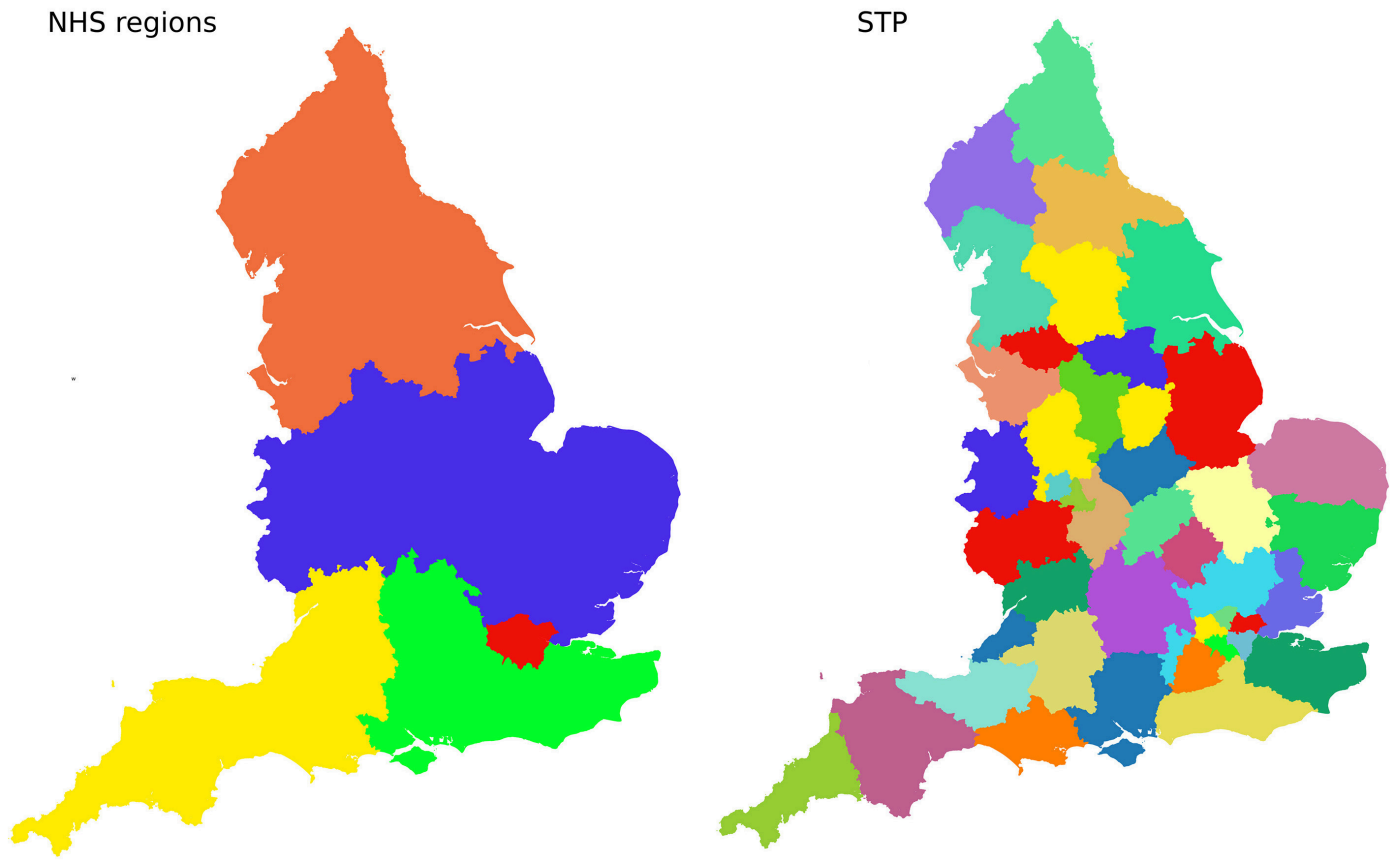
Boundaries of 5 NHS Regions **(Left)**, and 44 Sustainability and Transformation Partnerships (STPs, **Right**).

Figure 1 shows the geographical boundaries of STPs and NHS Regions.

### Optimizing Choice of Locations of Units

The model predicts, for any configuration of HASUs, the number of admissions to each HASU and the travel time from the patient's home to their closest (by travel time) HASU.

We used a bespoke genetic algorithm based on NSGA-II (14) to identify potential configurations of HASUs across England, balancing the competing objectives of access (travel time) and sustainability of expert services (admission numbers). We include any unit offering hyper-acute care (which will always include ability to treat with thrombolysis; units also offering thrombectomy are also considered HASUs for the purpose of this modeling).

In the algorithm, each solution is coded as binary array—with each element of that array corresponding to an existing stroke unit. A zero indicates that the unit is not selected to be a HASU, and 1 indicates that a unit is selected to be a HASU. The algorithm maintains a population of such solutions (typically, but not always, 10,000 solutions at any given time).These solutions go through a series of generations (typically 300–500). In each generation two existing solutions are selected and hybridized (with occasional random mutation). This is repeated until the “child” population (the new solutions) is as large as the “parent” population (the existing solutions). The two populations are combined and the best solutions are kept. For selection of best solutions we used a “pareto-based” method. Pareto selection is designed to capture all solutions that offer the best trade-off between competing multiple objectives. Solutions are eliminated if another solution is equally as good in all optimization parameters and is better in at least one parameter. The population may then be trimmed if necessary using “crowding distances” (where solutions that are very similar in results to others are more likely to be removed), or may be further expanded by repeating the pareto-selection process for the hitherto unselected solutions.

The selected configurations were based on a range of optimization parameters which seek to minimize travel times and to control admission numbers. These parameters were (1) number of HASUs (lower is better), (2) average travel time (lower is better), (3) maximum travel time (lower is better), (4) proportion of patients with a travel time of no more than 30 min (higher is better), (5) lowest number of admissions to any HASU (higher is better), (6) greatest number of admissions to any HASU (lower is better), (7) proportion of patients attending a HASU with at least 600 admissions per year (higher is better), (8) proportion of patients with a travel time of no more than 30 min and attending a HASU with at least 600 admissions per year (higher is better).

The algorithm was run assuming no boarders (patients may attend their closest HASU), or assuming patients may only attend a HASU within their own STP or NHS Region. Additional analysis was performed whereby solutions chosen assuming boarders were present, were then evaluated for performance if those boarders are then ignored for service delivery (patients may attend their closest HASU regardless of STP or NHS Region).

## Results

**Figure 2:**
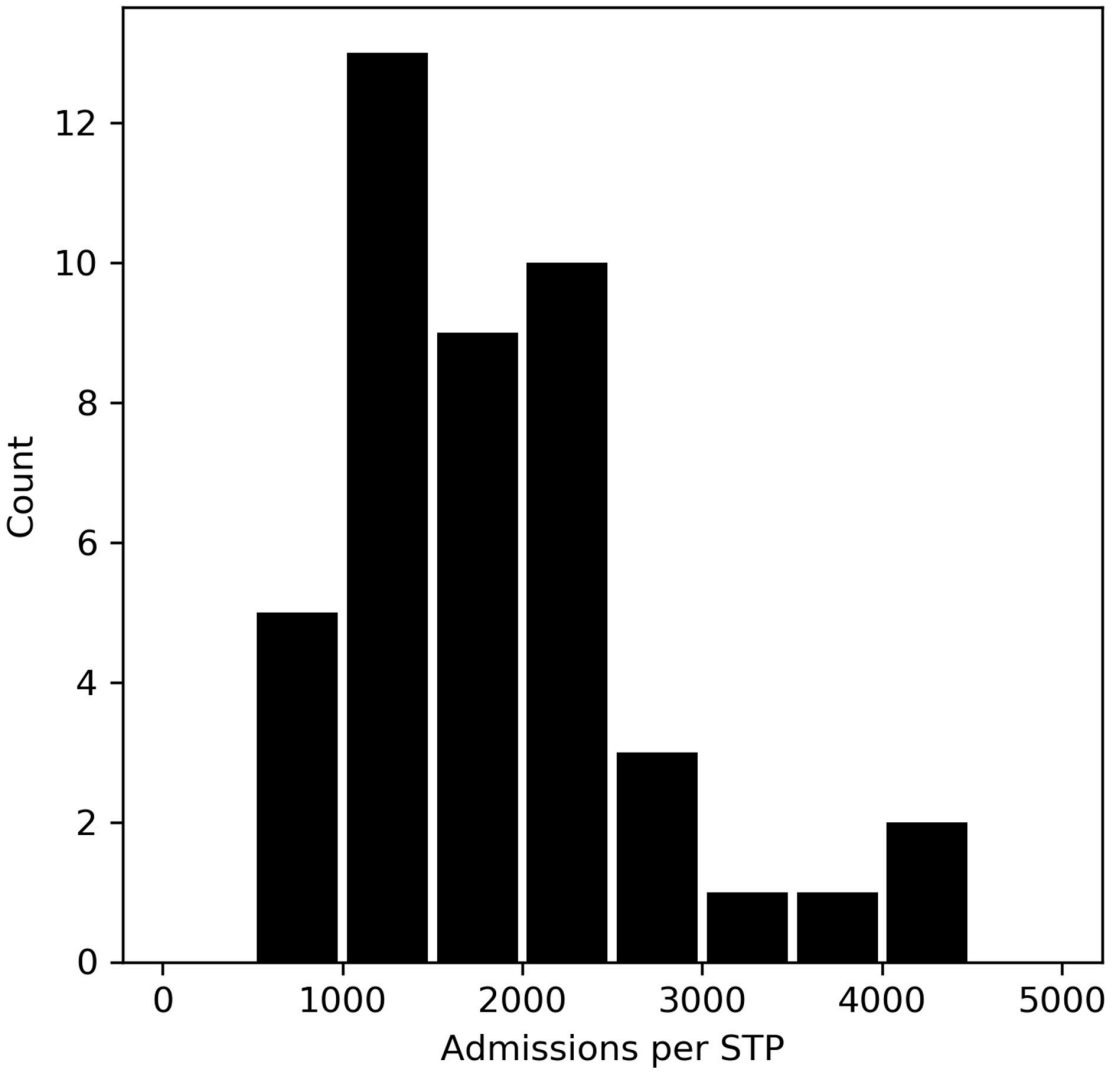
The number of stroke admissions per year by STP home location of patient. The x-axis shows admission numbers per STP in 500 admission bins, and the y-axis shows the count of STPs with that admission bin range.

Stroke admissions per STP ranged from 621 to 4,421 per year (Figure 2), a seven-fold range. Admissions per NHS Region range from 9,092 to 25,445.

**Figure 3:**
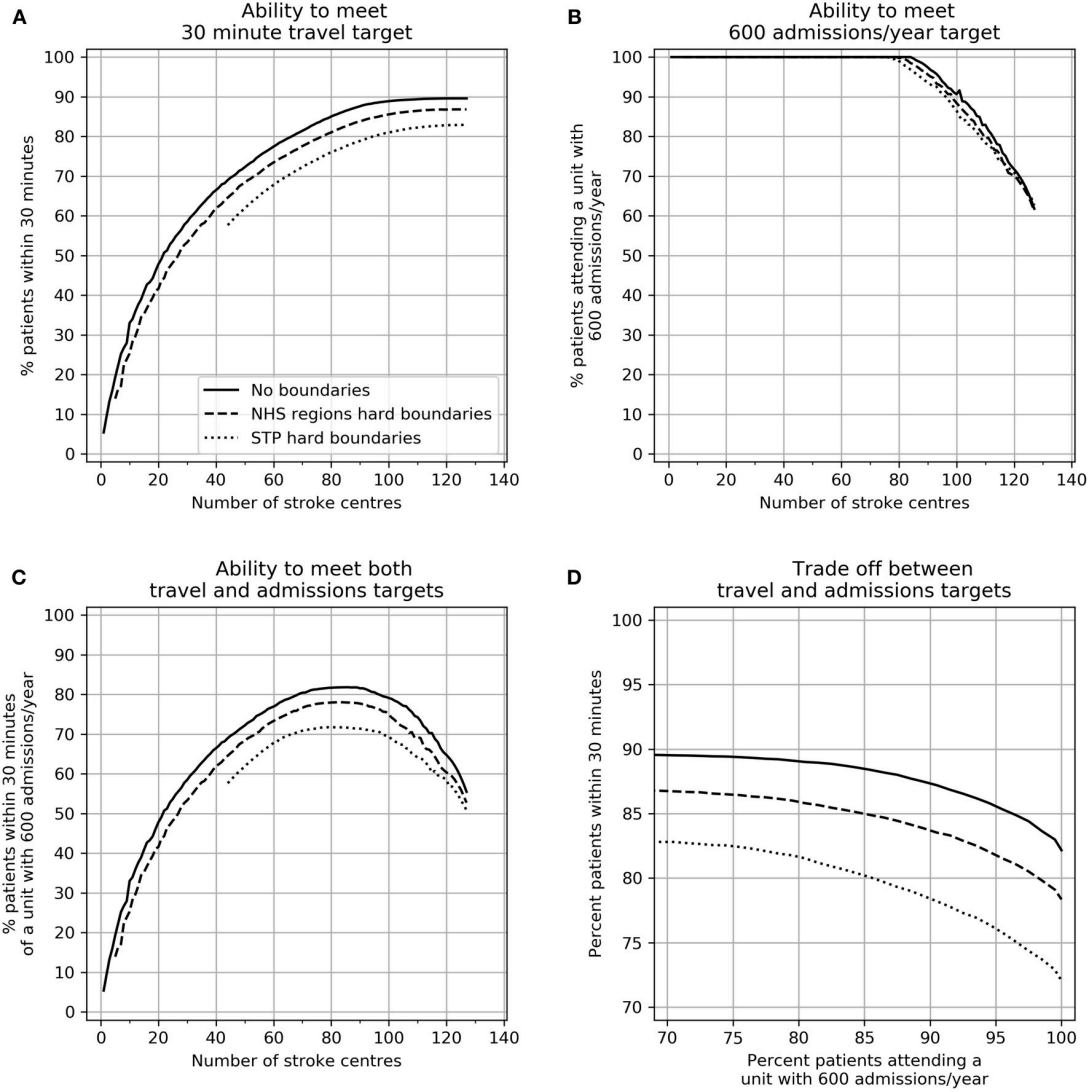
The effect of changing the number of HASUs and the planning footprint size on **(A)** the ability to provide 30 min access to HASUs, **(B)** the ability to meet a 600 admissions/year target, **(C)** the ability to meet both access and unit size targets, and **(D)** the best achievable compromise (Pareto front) between access and unit size targets. In all plots the solid line represents no boundaries, the dashed line restricts patients to NHS Region boundaries, and the dotted line restrict patients to STP boundaries.

The trade-off between achieving target admissions and target travel times are shown in Figure 3. These results assume that both planning and provision have hard boundaries (that is each region plans and provides for only their own constituents). Increasing the number of HASUs increases the proportion of patients within 30 min, however increasing the number of HASUs above about 80 leads to a decreasing proportion of patients being treated in units with at least 600 stroke admissions per year. The maximum proportion of patients attending HASUs that are both within 30 min and admit at least 600 stroke patients per year occurs at 70–90 HASUs nationally.

As planning footprint is reduced in size (from national, to NHS Regions, to STP) the ability to meet both target travel times and target admissions is compromised, though the effect on travel time is more pronounced. At all points the best achievable compromise between maximizing access (greatest number of patients within 30 min) and maximizing the number of patients attending a HASU with 600 admissions is compromised by planning within local or regional boundaries.

In order to meet stroke admission guidelines for all HASUs (600/year) the maximum possible proportion of patients within 30 min would be 82, 78, and 72% with no boundaries to planning/provision, NHS Region boundaries, and STP boundaries. The best achievable 95th percentile travel times would be 46, 47, and 55 min. To achieve this there would be 84, 79, or 76 HASUs for the three planning footprints.

We examined the effect on travel times and admission numbers if planning were performed at NHS Region or STP level, but then patients attended the closest HASU regardless of boundaries (closest by travel time). For these models we selected configurations that maximized the proportion of patients attending a HASU with target admission numbers, then selected configurations with the lowest maximum admission numbers (to control the size of the largest units), and then selected from those the configuration which maximized the proportion of people within 30 min of their closest planned HASU. These configurations had either 76 HASUs (optimization based on STPs) or 77 HASUs (optimization based on NHS Regions).

**Figure 4:**
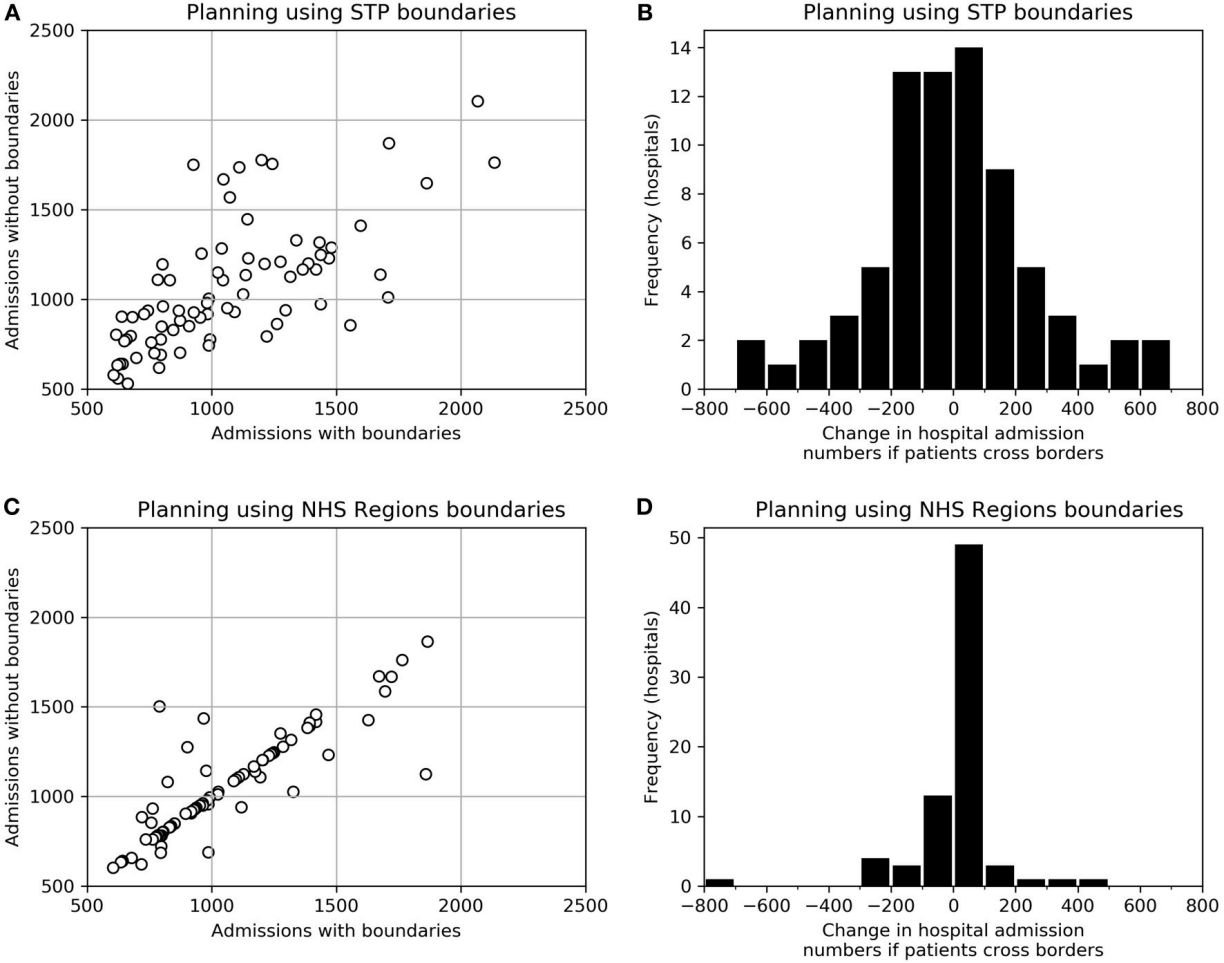
The effect of planning access to acute stroke services using STP or NHS Region boundaries, but then providing access without regard to boundaries. **(A)** Admission numbers to each HASU as originally optimized using STP boundaries, and admission numbers to the same HASUs if no boundaries are applied when providing care. **(B)** A histogram of the change in admission numbers between those expected when planning using STP boundaries and those expected if boundaries are ignored when providing care. **(C)** Admission numbers to each HASU as originally optimized using NHS Region boundaries, and admission numbers to the same HASUs if no boundaries are applied when providing care. **(D)** A histogram of the change in admission numbers between those expected when planning using NHS Region boundaries and those expected if boundaries are ignored when providing care.

When people attend their closest HASU regardless of boundaries, travel times are improved, compared with the original planned configuration assuming people stay within STPs or NHS Regions. When planning at NHS Region or STP level the proportion of patients within 30 min increased from 72–76 to 79% (for both planning levels). There is, however, significant disruption to admission numbers (Figure 4). When *planning* at STP level, assuming a contained population within a STP, but with *provision* then occurring without boundaries to people, admission numbers per HASU changed by an average of 204 (19%), and the number of HASUs achieving target admissions fell from 76 (all HASUs) to 73. The effect of planning at NHS Region level, but with patients then attending their closest HASU regardless of boundaries had significantly less effect. Admission numbers per HASU change by an average of 69 (7%), and all HASUs maintain the target 600 admissions per year.

## Discussion

Providing good access times to acute hospital services for the whole population necessarily requires a large number of hospitals. In contrast, providing 24/7 consultant-led services for time-critical emergency services (such as those for stroke) requires limits to be placed on the number of providers in order for each hospital to maintain target admissions required to sustain a specialist service that operates 24/7. These two objectives will always conflict, requiring careful judgement of the best balance between them. Computer modeling can help achieve a better, more quantitative, understanding of the trade-off between these conflicting objectives.

Currently, a little more than half of stroke patients attend a HASU with greater than the recommended 600 admissions per year; In 2015/16 61% of acute stroke admissions attended a HASU with at least 600 confirmed stroke admissions per year (2), with the largest unit having 2,001 admissions. Fifty out of 130 HASUs had at least 600 admissions per year. Fifty six percent of patients attend a unit with at least 600 admissions per year and have a travel time of no more than 30 min. Our modeling has previously suggested that the proportion of patients attending larger HASUs may be significantly increased with relatively minor changes to access times (11). However, the extended modeling described here suggests that achieving better access to larger HASUs is compromised if small geographical areas, such as STP footprints, are used to plan and provide these services. The main effect is on travel times; if patients cannot cross STP boundaries then they may be forced to travel further to a HASU.

Target travel times and minimum admission numbers can only ever be a guide. They may, however, help understand the nature and extent of the trade-offs required where it is not possible to achieve both objectives simultaneously. Modeling may help inform decision making by clarifying and quantifying those trade-offs.

A further challenge for organizing acute healthcare by STP footprint is the large variation in the number of people in STP areas, with the largest STP having seven times more admissions, than the smallest.

Though planning may occur at local STP level it is highly possible that those boundaries become ignored for provision, especially when considering where to take emergency stroke patients. The resulting discrepancy between planning footprint and the realities of provision is likely to make regional planning less accurate than it could be, especially concerning estimations of admission numbers, and also lead to a choice of service configurations that is ultimately sub-optimal both for patient travel times and for the ability to maintain target admissions required to sustain expert 24/7 services.

We have previously published work on optimizing access to thrombectomy services (12). A conclusion was that a sustainable and accessible acute stroke service will require a drip-and-ship approach, where patients first attend their closest HASU where they would have access to thrombolysis. They would then be transferred to a thrombectomy center if they were thought suitable and their most local acute stroke unit did not provide it. As it is the local acute stroke center that will be the first access point for stroke services we have focussed our work on the effect of regionalization on that first access point, that of the local hyper-acute stroke unit providing thrombolysis.

In this paper we have focussed on acute stroke care in England. The principles and methodology should be directly applicable to many other countries as well. Key principles are (1) to use a methodology that fully explores the range of compromises between time to access services, and the ability of those services to sustain 24/7 expert care, and (2) understand how regionalization of planning might be compromising performance of the system as a whole. The methods and code published may be directly transferred to other countries.

## Conclusions

Achieving best access times to acute hospital services for the whole population conflicts with the objective of reaching the minimum number of admissions in each type of unit required to provide good 24/7 consultant-led care for emergency or unplanned admissions. As the size of the geographical footprint for planning and provision is reduced to the level of STPs, the ability to meet access and admission targets becomes increasingly compromised, and the ability to predict admission numbers may be hampered by planning boundaries being ignored in practice. Planning and providing services at STP level could lead to sub-optimal service provision compared with using larger and more consistently populated planning areas.

Though we have focused on stroke care in England, the principles and methodology should be directly applicable to many other countries as well. Key principles are (1) to use a methodology that fully explores the range of compromises between time to access services, and the ability of those services to sustain 24/7 expert care, and (2) understand how regionalization of planning might be compromising performance of the system as a whole.

## Data Availability

All data, raw results, and optimization code used are available from the following repository: https://github.com/MichaelAllen1966/1807_acute_healthcare_location_effect_of_boundaries. The code runs in Python 3.6 (or higher), a free and open source programming language (see www.anaconda.com for a commonly used installation of Python complete with scientific libraries).

## Author Contributions

MA conceived the original research plan, contributed significantly to writing the optimization and analysis code, and wrote the first draft of the paper. KP contributed significantly to the writing of the optimization and analysis code and contributed amendments to the paper. EV wrote the original optimization code, and was the primary author of the technical description (Appendix in Supplementary Material). MJ has provided clinical oversight to the project, and contributed to the aims of the project, interpretation of results and writing of the paper. KS had overall oversight of the work and contributed to the aims of the project, interpretation of results, and writing of the paper.

### Conflict of Interest Statement

The authors declare that the research was conducted in the absence of any commercial or financial relationships that could be construed as a potential conflict of interest.

## References

[1] Feigin VL Forouzanfar MH Krishnamurthi R Mensah GA Connor M Bennett DA. Global and Regional Burden of Stroke during 1990-2010: findings from the Global Burden of Disease Study 2010. Lancet (2014) 383:245–55. 10.1016/S0140-6736(13)61953-4 24449944 PMC4181600

[2] Sentinel Stroke Audit Programme (SSNAP) Apr2015Mar2016-AnnualResultsPortfolio. National Results April 2015-Mar 2016. (2016). Available online at: https://Www.Strokeaudit.Org/Documents/Results/National/Apr2015Mar2016/Apr2015Mar2016-AnnualResultsPortfolio.Aspx 2016

[3] Newton JN Briggs ADM Murray CJL Dicker D Foreman KJ Wang H. Changes in health in England, with analysis by English Regions and Areas of Deprivation, 1990-2013: a systematic analysis for the Global Burden of Disease Study 2013. Lancet (2015) 386:2257–74. 10.1016/S.0140-6736(15)00195-6 26382241 PMC4672153

[4] Morris S Hunter RM Ramsay AIG Boaden R McKevitt C Perry C. Impact of centralising acute stroke services in English metropolitan areas on mortality and length of hospital stay: difference-in-differences analysis. BMJ (2014) 349:g4757. 10.1136/bmj.g4757 25098169 PMC4122734

[5] Hunter RM Davie C Rudd A Thompson A Walker H Thomson N. Impact on clinical and cost outcomes of a centralized approach to acute stroke care in London: a comparative effectiveness before and after model. PLoS ONE (2013) 8:1–9. 10.1371/journal.pone.0070420. 23936427 PMC3731285

[6] NHS England Putting Patients First: The NHS Business Plan 2013/4 - 2015/6. (2013). Available online at: https://www.england.nhs.uk/wp-content/uploads/2013/04/ppf-1314-1516.pdf

[7] Price C James M Meeting the Future Challenge of Stroke Meeting the Future Challenge of Stroke. British Association of Stroke Physicians. (2011). Available online at: https://basp.ac.uk/wp-content/uploads/2017/02/BASP-Meeting-the-Future-Challenge-of-Stroke-2011-15.pdf

[8] NHS England Stroke Services: Configuration Decision Support Guide (2015).

[9] NHS Delivering the Forward View: NHS Planning Guidance 2016/17 – 2020/21 (Gateway Reference 04437). (2015). Available online at: https://web.archive.org/web/20180406041635/https://www.england.nhs.uk/wp-content/uploads/2015/12/planning-guid-16-17-20-21.pdf

[10] NHS England Our Business Plan 2016/17. (2016). Available online at: https://www.england.nhs.uk/wp-content/uploads/2016/03/bus-plan-16.pdf

[11] Allen M Pearn K Villeneuve E Monks T Stein K James M Feasibility of a hyper-acute stroke unit model of care across England. A modelling analysis. BMJ Open (2017) 17:12 10.1136/bmjopen-2017-018143 PMC5736033 29247093

[12] Allen M Pearn K James M Ford GA White P Rudd AG Maximising access to thrombectomy services for stroke in England: a modelling study. Eur Stroke J. (2018). 10.1177/2396987318785421. [Epub ahead of print]. PMC6533864 31165093

[13] Consumer Data Research Centre CDRC 2011Population Weighted Centroids - GB 2015. (2015). Available online at: https://data.cdrc.ac.uk/dataset/cdrc-2011-population-weighted-centroids-gb

[14] Deb K Pratap A Agarwal S Meyarivan T A fast and elitist multiobjective genetic algorithm: NSGA-II. IEEE Trans Evol Comput. (2002) 6:182–97. 10.1109/4235.996017

