# Supplementary material for: Planning and Providing Acute Stroke Care in England: The Effect of Planning Footprint Size
Source: Front Neurol. 2019 Feb 27;10:150. doi: 10.3389/fneur.2019.00150 (PMC6400850; doi:10.3389/fneur.2019.00150)
Supplement: Supplementary file 1 [file Data_Sheet_1.pdf]

Appendix

1 Description of problem

In order to establish a hyper-acute stroke unit (HASU) model for emergency stroke care across England, all HASUs should have a minimum of 600 yearly admissions of confirmed strokes. No unit should be infeasibly large (and we have taken the current largest unit with ~2,000 stroke admissions per year as our upper target). All patients are expected to be taken to their closest HASU, with ‘closest’ chosen by estimated road travel times.

The problem involves looking for solutions that can place any number of hospitals in any of 127 locations. There are therefore 2^127 or 10^38 possible solutions. Each solution requires looking up road travel times from each of 32,843 patient locations to all open hospitals to allocate patients to their closest hospital. There are 13 possible objectives to achieve or trade-off (see section 4.1).

This type of problem is termed ‘NP-hard’ - it cannot be solved explicitly in reasonable time. And as there are multiple-objectives that trade-off against each other there is no single solution to the problem (as there is no way to objectively determine the weighting of different objectives); rather we are looking for a population of solutions which demonstrate the trade-off between different objectives.

With NP-hard problems there are often a range of different heuristic algorithms which search for good solutions to the problem, while never guaranteeing an optimal solution is found. One set of general purpose heuristic methods are a family of algorithms known as ‘genetic algorithms’, due to their inspiration coming from the theory of evolution. Here we describe the specific genetic algorithm used in our study.

2 NHS planning boundaries

Fig 1 shows the five NHS Regions and 44 Sustainability and Transformation Partnerships (STPs) planning footprints studied.

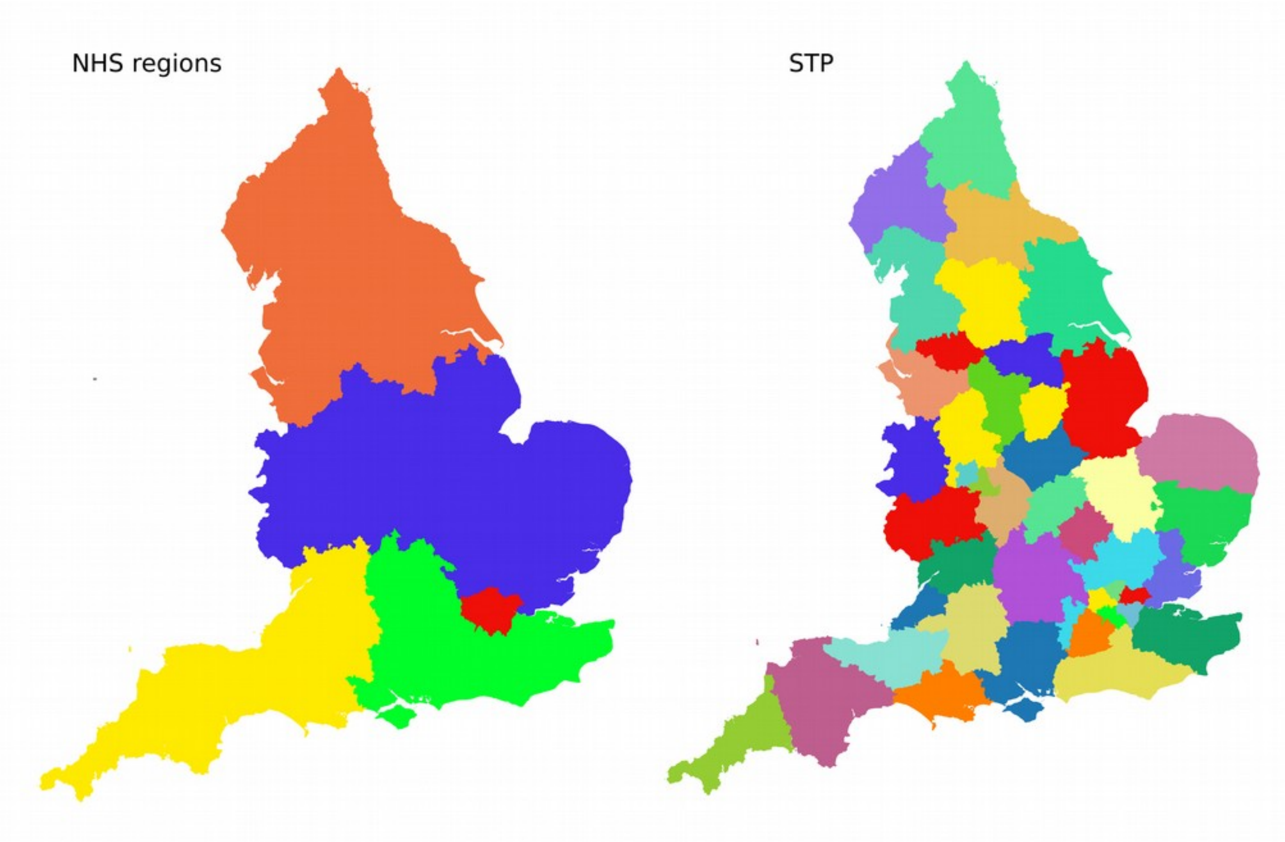

Figure 1: Boundaries of 5 NHS Regions (left), and 44 STPs (right).

The algorithm could be performed according to three boundary rules:

1. Patients may attend any hospital (and attend the closest)
2. Patients may only attend hospitals in the NHS Region where they live (they attend the closest hospital in the region)
3. Patients may only attend hospitals in the STP where they live (they attend the closest hospital in the STP)

3 Code and data repository

Data, code used for the model, and all raw results are available at:

https://github.com/MichaelAllen1966/1807_acute_healthcare_location_effect_of_boundaries

Note: The code contains a bespoke Genetic Algorithm written in Python/NumPy.

4 Multi-objective problem

4.1 Pareto dominance

When solving an optimisation problem based on one objective, the optimal solution is given by the configuration with the best (highest or lowest) objective value. In the case of multi-objective optimisation, comparing several solutions requires to reference to the notion of dominance: a vector

a of the objective space dominates another vector *b* if all criteria of *a* are better or equal to criteria of *b* and $a \neq b$ [1]. Then, a solution is non-dominated if there are no other solutions at least equal in all objectives and better in at least one objective. At the end of the optimisation process, there is no single best solution but a set of non-dominated solutions, called the Pareto Front. An example of a Pareto Front using two objectives is shown in figure 2.

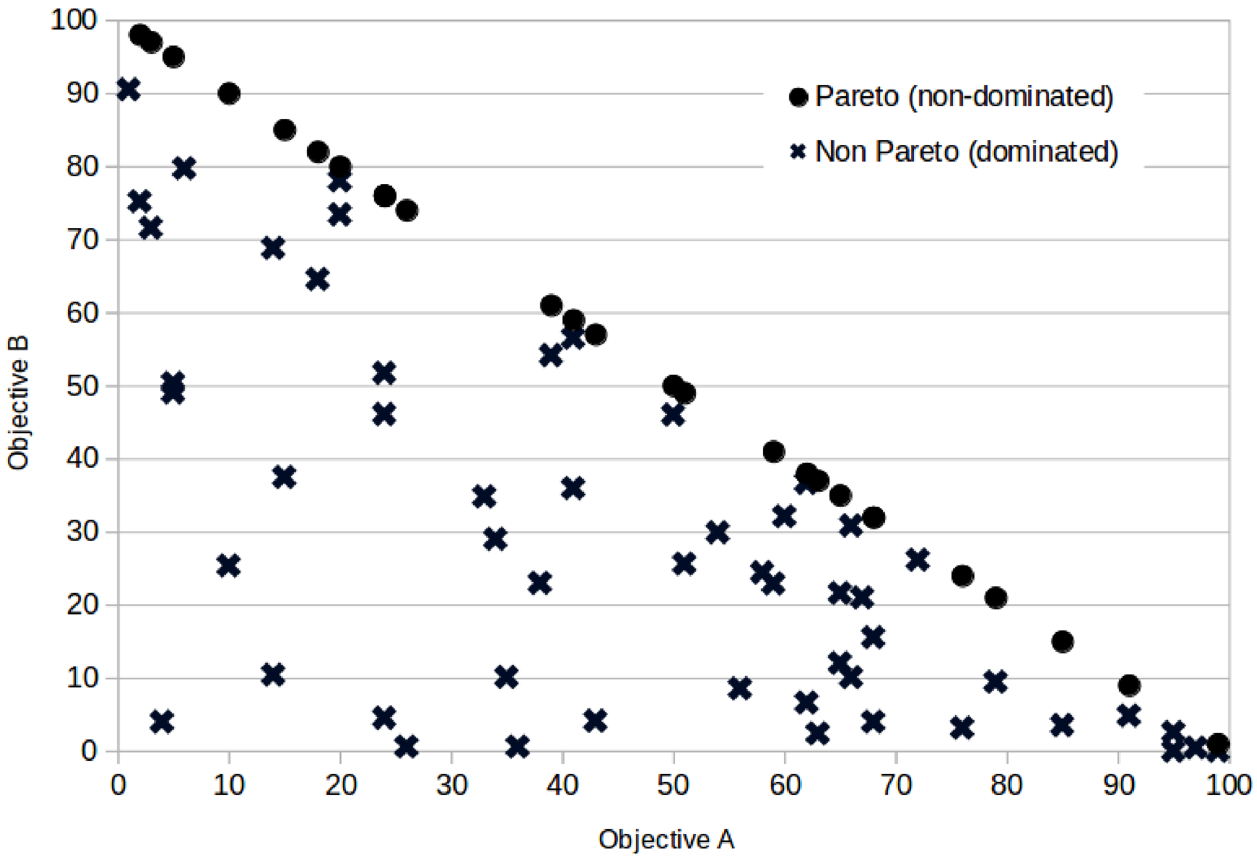

Figure 2: Example of identification of Pareto front (non-dominated) points when comparing two objectives.

The greater the number of objectives on the Pareto Front the lower the chance that a point will be dominated by another. If there is no correlation between objectives and solutions are entirely random then the chance of a single point being dominated by another single point picked at random is $0.5^\wedge n_{obj}$ (where n_{obj} is the number of objectives).

4.2 Algorithm objectives

The objectives which could be used to select solutions were:

1: Number of hospitals (lower is better)

2: Average travel time (lower is better)

3: Maximum travel time (lower is better)

4: Maximum admissions to any one hospital (lower is better)

5: Minimum admissions to any one hospital (higher is better)

6: Max/Min admissions ratio (lower is better)

7: Proportion patients within estimated 30 min travel distance (higher is better)

8: Proportion patients within estimated 45 min travel distance time (higher is better)

9: Proportion patients within estimated 60 min travel distance time (higher is better)

10: Proportion patients attending unit with target admission numbers (higher is better)

11: Proportion patients attending unit with target admission numbers and within estimated 30 min travel time (higher is better)

12: Proportion patients attending unit with target admission numbers and within estimated 45 min travel time (higher is better)

13: Proportion patients attending unit with target admission numbers and within estimated 60 min travel time (higher is better)

The algorithm allows restriction of choice to local regions if needed, in which case patients may only attend a hospital in their own local region. These solutions may be later re-evaluated using no restrictions.

Attempting to optimise on all 13 objectives simultaneously produces very slow progress. The progressive extension of objectives is an established general methodology for genetic algorithms [2].

The advantage of the smaller objective sets is that the chance of Pareto dominance is greater (see section 4.1), leading to greater selection pressures in the algorithm. As an example when starting with a random population of 10,000 solutions the proportion of solutions in the first generation (the randomly chosen generation) that were on the Pareto Front were as follows:

All objectives: Mean 3,295 solutions on Pareto Front (SD =115, n=5)

3 Objectives (8,10,12): Mean 49 solutions on Pareto Front (SD =7, n=5)

Initial runs (with no boarders, NHS Region boarders, or STP boarders) were focussed on core objectives 1, 2, 4 & 5. The first run composed of 500 generations with a minimum population size of 5,000 and a maximum population size of 10,000 (see section 5.6). After this all objectives were introduced and the algorithm run with no limitation to population size, but with the algorithm being stopped when population size reached 50,000.

5 Genetic algorithms

Genetic algorithms manage a population of individuals encoded as vectors through a given number of generations. At each generation, ‘good’ parents are selected from the population according to their fitness (any measure of superiority over other potential parents). Parents are then combined, using a cross-over operator, to create children which are finally mutated. Genetic algorithms differ in the parent selection process, in the cross-over and mutation processes, and in the way the population is archived.

5.1 Representation

Solutions are coded as binary string of genes with either 1 for an open location or 0 for closed. For instance, 001011 would be six genes that represent hospitals 3, 5 and 6 being open and 1, 2 and 4 being closed. In this study, vectors represent the 127 hospitals (SSNAP acute admitting stroke units).

5.2 Selection

The selection operator chooses a part of the population to become parents. The better individuals in terms of objective values are more likely to become parents. The selection probability can be proportionate to fitness by roulette-wheel sampling [3] or stochastic universal sampling [4]. The sigma scaling method normalises the fitness by its variance in the population, so that the individuals with the highest fitness always have a higher probability than others to produce children. However, these approaches focus on exploitation of existing population rather than exploration of the decision space and they can lead to premature convergence.

Other selection methods rely on ranking rather than fitness value. With ranking selection, individuals are ranked according to their fitness and their probability to become parents is function of their rank [5]. Similarly, the tournament selection creates random pairs of individuals and keeps the one with the highest fitness value with a given probability [3]. Such methods allow the algorithm to keep some individuals with low fitness values (with the advantage of keeping a broader gene pool).

Finally, the Boltzmann selection [6] controls the selection rate via a temperature. At the beginning, all individuals have a similar probability to be selected. As the temperature decreases, the selection focusses on high-fitness individuals.

5.3 Cross-over

The cross-over is the process which exchanges genes from parents to create new children. The simplest option is the single-point cross-over which selects one locus and exchanges the blocks of parents before and after that locus. For instance, a crossover at point five would perform the following:

Parent A:	1 1 1 1 1 1
Parent B:	0 0 0 0 0 0
Child A: =	1 1 1 1 0 0
Child B:	0 0 0 0 1 1

The choice of the single-point location can be made by a uniform distribution. In the case of binary vectors, the single-point cross-over is less likely to exchange the endpoints of vectors [2]. To reduce this effect, the cross-over can rely on two or more exchange points.

5.4 Mutation

Mutation changes the gene value of each locus, with a very small probability for each individual each generation. According to [7], the mutation process avoids the loss of diversity in the population.

5.5 Archive

Genetic algorithms also vary by the way solutions are archived and if the population size is variable. The simple option is to keep only children. However, it assumes that children are better than parents which are lost. Several methods build an archive which is union of parents and children. If the population size is variable, an option is to keep the Pareto Front of this archive. However, the size of this Pareto Front can increase dramatically, in particular with many objective functions. Then, individuals from the archive are ranked, based on their Pareto dominance and another metric. NSGA-II [8] and SPEA2 [9] both rank individuals by combining dominance and spread metric in order to maximise population diversity.

5.6 The NSGA-II method

In NSGA-II [8] the archive and the new population are merged and all individuals are ranked according to a two-step mechanism. In the first step, the merged population is split into layers of non-dominated fronts, the first layer being the Pareto Front (the second layer being the next Pareto Front after removal of the first layer). In the second step, the spread of the population is measured by the crowding distance which gives the distance from an individual to its nearest neighbour. To keep the size of the population constant, a given number of individuals is selected from the merged population, preferably from the upper layers and with the largest crowding distance.

NSGA-II has the advantage to keep not only optimal solutions but also near-optimal solutions in lower layers. However, to do so, the population must be large enough. The second advantage is to provide a diverse population in terms of score values, thanks to the crowding distance ranking.

The NSGA-II was chosen for this study after a pilot comparison with SPEA2 [9], MOEAD [10], and HypE [11] which showed that NSGA-II provided similar objective performances with a more diverse population.

5.7 Convergence indicator

Population diversity can be monitored using average Hamming distance. The Hamming distance between any two solutions is the proportion of genes that are different. Average Hamming distance is the mean Hamming distances for all pairwise comparisons in the population (after first Pareto Front selection).

5.8 Description of our genetic algorithm

The code contains a bespoke implementation of a genetic algorithm, based on NSGA-II [8]. Our method evolves solutions based on multiple objectives, but without any weighting of objectives. In each generation, the Pareto Front of non-dominated solutions is identified. Larger populations may

be selected by picking subsequent Pareto Fronts (re-evaluation the Pareto Front after removal of the previous Pareto Front identified). The population size is maintained in the interval $\left[P_{min}; P_{max}\right]$.

The steps of the algorithm are:

1) Identify which combination of objectives to use for selection in algorithm (may be from 2 objectives to all objectives).

2) Set up initial population of solutions (a typical starting population is 10,000 solutions).

i) Randomly choose number of hospitals to open in each solution.

ii) Randomly assign open hospitals.

iii) A library of solutions may be imported instead of, or in addition to, a random population of solutions.

iv) Non-unique solutions are removed.

3) Breed solutions:

i) Choose pairs of solutions at random from the population.

While NSGA-II selects parents with the tournament method based on weighted criteria, our method selects parents randomly to avoid weighting any objective.

ii) Select a single crossover point at random within the solution binary string.

iii) Apply the cross-over operator to produce children.

iv) Randomly mutate children with a probability per element of 0.005.

v) Combine parents and children into a new population.

vi) Remove non-unique solutions and any solutions where all hospitals are closed.

4) Calculate the performance of all solutions against the objectives used for selection.

5) Identify all non-dominated (Pareto Front) solutions

i) If the number of selected solutions is greater than the maximum permitted population size then reduce the number of solutions by either

(1) picking the required number of solutions at random, or

(2) pick two solutions at random and use tournament selection based on crowding distance

ii) If the number of selected solutions is lower than the target population then remove the previously selected non-dominated solutions and repeat the Pareto selection until sufficient solutions have been identified.

6) Add in an extra 5% of new random solutions

7) Repeat steps 3-5 until the maximum number of generations is reached or the algorithm is stopped by another indicator:

Note: The minimum and maximum number of solutions to pass on to the next generation may be the same number to keep solution size constant. Alternatively, a range of population size may be acceptable (e.g. a minimum number of 1,000 solutions may be chosen, but a maximum number of 5,000 solutions may be permitted. In this case Pareto selection is repeated until at least 1,000 solutions have been selected, but restriction on the number of solutions only occurs if the number of solutions chosen exceeds 5,000).

6 References

1 Zhou A, Qu B-Y, Li H, *et al.* Multiobjective evolutionary algorithms: A survey of the state of the art. *Swarm Evol Comput* 2011;**1**:32–49. doi:10.1016/j.swevo.2011.03.001

2 Mitchell M. An introduction to genetic algorithms. *Comput Math with Appl* 1996;**32**:133. doi:10.1016/S0898-1221(96)90227-8

3 Goldberg DE. *Genetic Algorithms in Search, Optimization, and Machine Learning*. 1989. doi:10.1007/s10589-009-9261-6

4 Baker JE. Reducing bias and inefficiency in the selection algorithm. *Proc Second Int Conf Genet Algorithms Genet algorithms their Appl* 1987;:14–21.

5 Baker JE. Adaptive selection methods for genetic algorithms. *Proc an Int Conf Genet Algorithms their Appl* 1985;:101–11.

6 Maza MDA, Tidor B. An analysis of selection procedures with particular attention paid to proportional and Boltzmann selectiono Title. *Proc 5th Int Conf Genet Algorithms* 1993;:124–31.

7 Holland JH. *Adaptation in Natural and Artificial Systems: An introductory Analysis with Applications to Biology, Control and Artificial Intelligence*. 1975. doi:10.1137/1018105

8 Deb K, Pratap A, Agarwal S, *et al.* A fast and elitist multiobjective genetic algorithm: NSGA-II. *IEEE Trans Evol Comput* 2002;**6**:182–97. doi:10.1109/4235.996017

9 Zitzler E, Laumanns M, Thiele L. SPEA2: Improving the Strength Pareto Evolutionary Algorithm. *Evol Methods Des Optim Control with Appl to Ind Probl* 2001;:95–100. doi:10.1.1.28.7571

10 Zhang Q, Li H. MOEA/D: A multiobjective evolutionary algorithm based on decomposition. *IEEE Trans Evol Comput* 2007;**11**:712–31. doi:10.1109/TEVC.2007.892759

11 Bader J, Zitzler E. HypE: An Algorithm for Fast Hypervolume-Based Many-Objective Optimization. *Evol Comput* 2011;**19**:45–76. doi:10.1162/EVCO_a_00009
